# Cardiac Beriberi After Gastrectomy Presenting With Peripheral Neuropathy and Mimicking Tachycardia-Induced Cardiomyopathy: A Case Report

**DOI:** 10.7759/cureus.91307

**Published:** 2025-08-30

**Authors:** Satoshi Kurisu, Hitoshi Fujiwara

**Affiliations:** 1 Department of Cardiology, National Hospital Organization (NHO) Hiroshima-Nishi Medical Center, Otake, JPN

**Keywords:** alcohol use, atrial fibrillation, atrial fibrillation (af), food and nutrition, gastrectomy, heart failure

## Abstract

Cardiac beriberi, a manifestation of thiamine deficiency, is an uncommon but reversible cause of heart failure that can mimic other forms of cardiomyopathy, such as dilated or tachycardia-induced cardiomyopathies. It is likely underrecognized or misdiagnosed. We report an elderly man with neurological symptoms and globally reduced left ventricular (LV) function in the context of prior gastrectomy, chronic alcohol consumption, and prolonged malnutrition. Early empiric thiamine administration led to rapid improvement in pulse rate, left ventricular function, and neurological symptoms, and the diagnosis was confirmed by a low thiamine level. This case underscores the importance of considering cardiac beriberi in the differential diagnosis of newly diagnosed heart failure, particularly in patients with nutritional risk factors or a history of gastrointestinal surgery. Early recognition and treatment are crucial to preventing adverse outcomes.

## Introduction

Thiamine (vitamin B1) is a low-molecular-weight, water-soluble, sulfur-containing aminopyridine essential for energy metabolism [1]. Thiamine deficiency can result in diverse clinical manifestations, which are generally classified into two major clinical forms. The first primarily affects the nervous system and is known as dry beriberi, while the second involves the cardiovascular system and is referred to as wet beriberi. Dry beriberi can present as peripheral neuropathy or Wernicke’s encephalopathy [2-4]. In contrast, wet beriberi typically presents as chronic right-sided heart failure with high cardiac output [5,6]. Shoshin beriberi is a fulminant form of cardiac beriberi, characterized by an acute onset of hypotension, tachycardia, and lactic acidosis [7,8]. Although it usually manifests with a hyperdynamic circulatory state, some cases present with extremely low cardiac output [7,8], in marked contrast to the typical early high output state.

Cardiac beriberi, although uncommon in developed countries, remains a clinically significant and potentially reversible cause of heart failure. Established risk factors include chronic alcohol consumption, prolonged malnutrition, and prior gastrointestinal surgery associated with impaired long-term thiamine absorption [8,9]. Cardiac beriberi is likely underrecognized or misdiagnosed because its clinical presentation can resemble other forms of cardiomyopathy, such as dilated or tachycardia-induced cardiomyopathies [10]. Early recognition of cardiac beriberi remains crucial due to the potential reversibility of the condition with appropriate thiamine replacement.

Here, we report a case of cardiac beriberi in a patient with a remote history of gastrectomy, whose clinical presentation initially mimicked tachycardia-induced cardiomyopathy [11,12] or hyperthyroidism with significant cardiac involvement [13,14].

## Case presentation

A 79-year-old man with a history of gastrectomy for gastric cancer 45 years earlier and a remote history of pulmonary tuberculosis presented to a local clinic with generalized fatigue, palpitations, shortness of breath, and progressive bilateral lower extremity weakness that led to difficulty walking. Tachycardia was noted, and he was referred to our hospital for further evaluation. He had not been receiving regular medical care and was not on any prescribed medications.

On physical examination, his pulse rate was irregular at 160 bpm on the monitor; blood pressure, 78/50 mmHg; oxygen saturation, 96%; body weight, 39 kg; and body mass index, 15.4 kg/m^2^. Manual muscle testing revealed a score of 2 out of 5 in the lower extremities, indicating an inability to move against gravity. Mild edema was observed in both lower extremities. He reported drinking about 1 L of beer daily and consuming a chronically unbalanced diet consisting mainly of instant noodles.

Blood tests revealed normal liver, renal, and thyroid function (Table 1). However, the N-terminal pro-brain natriuretic peptide (NT-proBNP) level was markedly elevated to 1,978 pg/mL (reference range: <126 pg/mL).

**Table 1:** Laboratory data Blood tests revealed normal liver, renal, and thyroid function. However, the NT-proBNP level was markedly elevated to 1,978 pg/mL. IU: international units, NT-proBNP: N-terminal pro-brain natriuretic peptide

Variable	Day 1	Day 9	Reference range
Blood count			
White blood cell count (/µL)	8.1 × 10^3^	3.8 × 10^3^	3.3-8.6 × 10^3^
Red blood cell count (/µL)	4.13 × 10^6^	3.63 × 10^6^	4.35-5.55 × 10^6^
Hemoglobin (g/dL)	13.9	12.3	13.7-16.8
Hematocrit (%)	41.0	37.5	34.9-45.1
Mean corpuscular volume (fL)	99.3	103.3	83.6-98.2
Platelet count (/µL)	168 × 10^3^	234 × 10^3^	158-348 × 10^3^
Blood chemistry			
Aspartate aminotransferase (U/L)	18	37	13-30
Alanine aminotransferase (U/L)	7	27	10-42
Lactate dehydrogenase (U/L)	245	217	124-222
Blood urea nitrogen (mg/dL)	23.1	14.1	8-20
Creatinine (mg/dL)	0.72	0.68	0.65-1.07
Estimated glomerular filtration rate (mL/min/1.73 m^2^)	79.3	84.4	-
Sodium (mmol/L)	140	136	138-145
Potassium (mmol/L)	3.85	4.52	3.6-4.8
Chloride (mmol/L)	103	103	101-108
Creatine phosphokinase (U/L)	161	41	59-248
Total cholesterol (mg/dL)	216	-	142-248
Glucose (mg/dL)	131	-	73-109
C-reactive protein (mg/dL)	0.14	0.05	<0.14
NT-proBNP (pg/mL)	1,978	673	<18.4
Thyroid-stimulating hormone (µIU/mL)	3.63	-	0.61-4.23
Free thyroxine (ng/dL)	0.96	-	0.7-1.48
Thiamine (vitamin B1) (ng/mL)	15	138	24-66
Venous gas analysis			
pH	7.428	-	7.35-7.45
HCO_3_^-^ (mmol/L)	27.6	-	22.2-28.3
Lactate (mmol/L)	2.0	-	0.5-1.6

**Figure 1:**
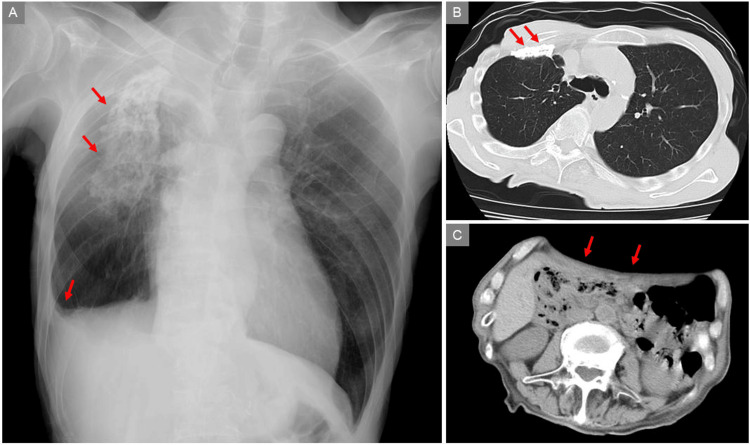
Chest radiography and computed tomography images Chest radiography showed calcifications in the right upper and middle lung fields and blunting of the right costophrenic angle (A, arrows). Computed tomography revealed calcifications along the anterior pleura without pleural effusion (B, arrows), and these findings were considered sequelae of prior pulmonary tuberculosis. A concave abdominal wall was also noted (C, arrows), suggesting malnutrition or loss of subcutaneous fat.

Chest radiography showed calcifications in the right upper and middle lung fields and blunting of the right costophrenic angle (Figure 1A, arrows). Computed tomography (CT) revealed calcifications along the anterior pleura without pleural effusion (Figure 1B, arrows), and these findings were considered sequelae of prior pulmonary tuberculosis. A concave abdominal wall was also noted (Figure 1C, arrows), suggesting malnutrition or loss of subcutaneous fat.

**Figure 2:**
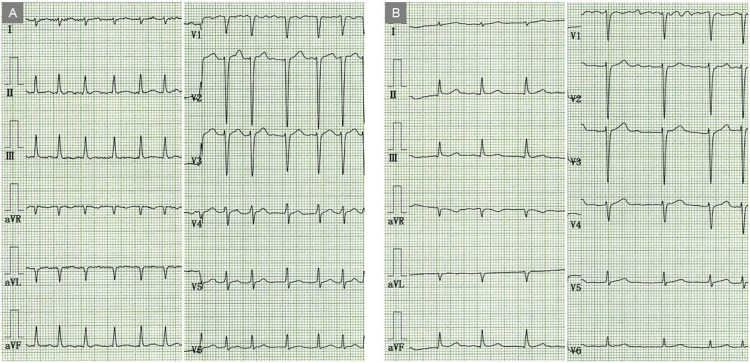
Electrocardiograms at presentation and on hospital day 7 At presentation, electrocardiography revealed atrial fibrillation with an irregular RR interval and a rapid ventricular response (158 bpm) (A). On hospital day 7, the patient remained in atrial fibrillation, but the pulse rate had stabilized at 84 bpm (B).

Electrocardiography (ECG) revealed atrial fibrillation with an irregular RR interval and a rapid ventricular response (158 bpm) (Figure 2A).

**Figure 3:**
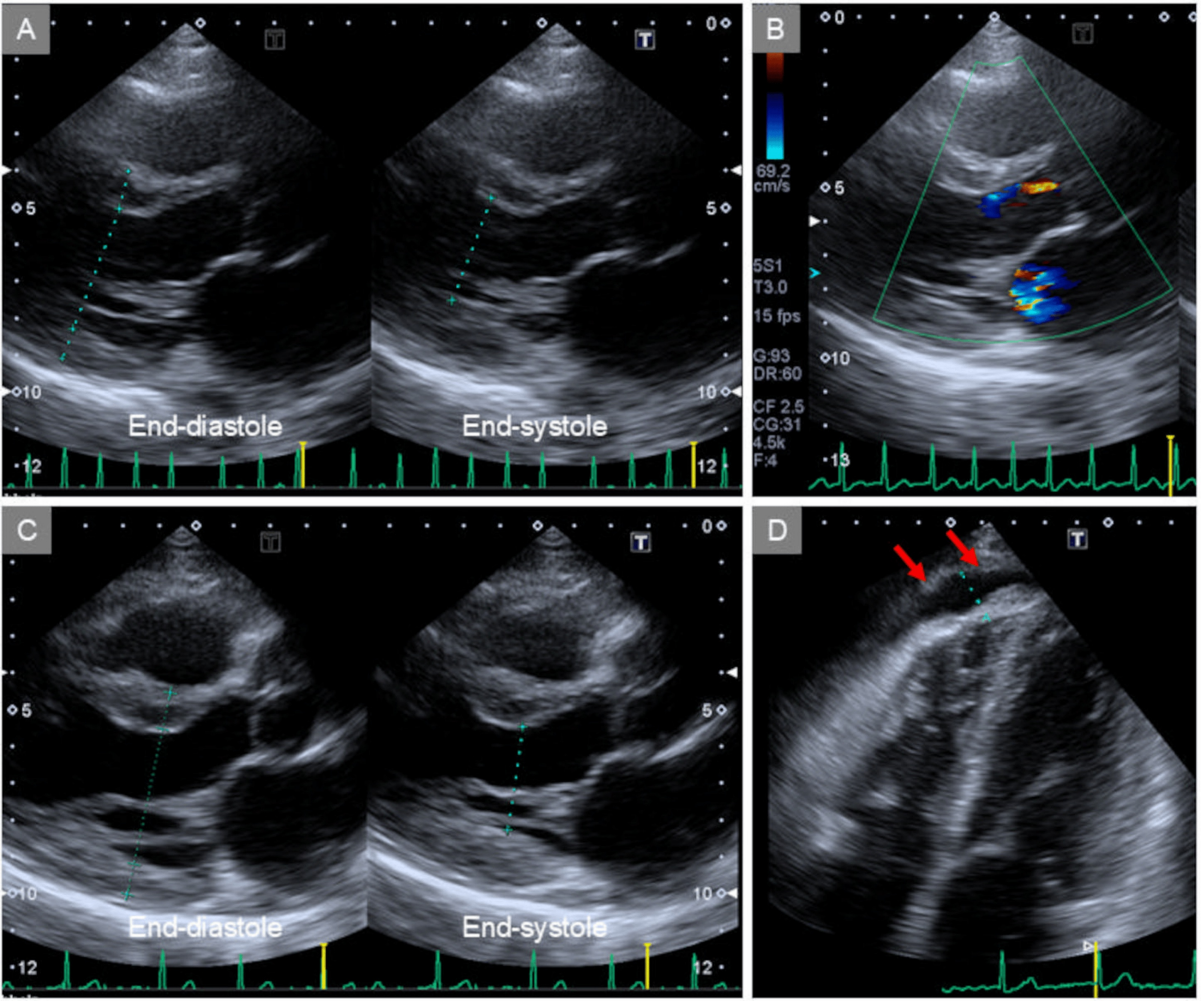
Echocardiographic images at presentation and on hospital day 7 At presentation, transthoracic echocardiography revealed globally reduced LV systolic function, with an EF of approximately 30% (A). Moderate mitral regurgitation was noted (B). On hospital day 7, repeated echocardiography demonstrated marked improvement in LV function over a short period, with an EF of 60% (C). A small amount of pericardial effusion remained (D, arrows). LV: left ventricular, EF: ejection fraction

Transthoracic echocardiography revealed globally reduced left ventricular (LV) systolic function, with an ejection fraction (EF) of approximately 30% (Figure 3A). The LV internal dimension and wall thickness were within normal limits. Moderate mitral regurgitation (Figure 3B) and a small pericardial effusion were noted. The patient was admitted for treatment and further cardiac evaluation.

**Figure 4:**
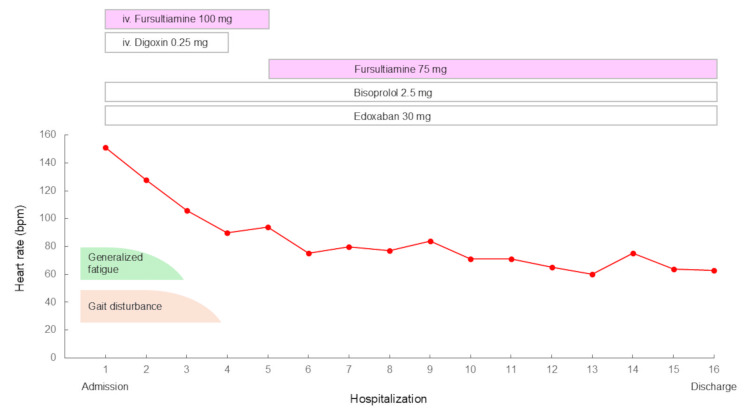
Clinical course during hospitalization Empiric intravenous thiamine therapy was started after obtaining samples for thiamine level measurement. iv.: intravenous

Given the constellation of peripheral neuropathy and the patient’s history of gastrectomy, chronic alcohol consumption, and unbalanced dietary habits, beriberi was suspected. Empiric intravenous thiamine therapy (fursultiamine 100 mg/day) was started after obtaining samples for thiamine level measurement (Figure 4). Rate control was initiated with oral bisoprolol and intravenous digoxin for rapid atrial fibrillation with reduced LV function. After three days of treatment, including intravenous thiamine and physical rehabilitation, his generalized fatigue and lower extremity weakness markedly improved, and he regained the ability to walk independently. His pulse rate gradually decreased to below 100 bpm. Given the predisposition to thiamine deficiency following gastrectomy, intravenous thiamine was switched to oral administration (fursultiamine 75 mg/day) for maintenance therapy. Subsequently, the thiamine level measured before treatment was found to be 15 ng/mL (reference range: 24-66 ng/mL), confirming the diagnosis of beriberi.

**Figure 5:**
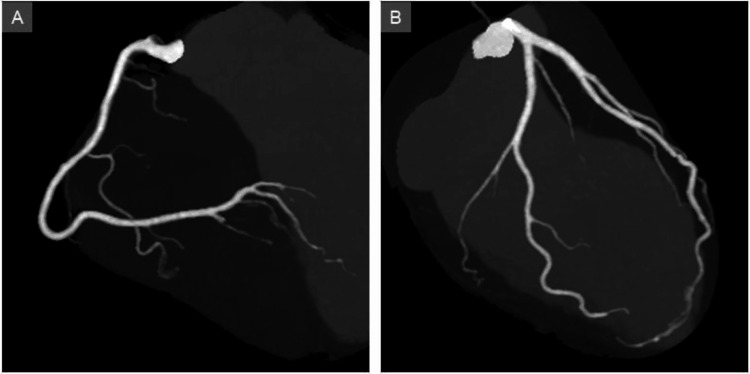
Computed tomography angiography Computed tomography angiography revealed no significant stenosis in either the right or the left coronary artery (A and B).

On hospital day 7, the patient remained in atrial fibrillation, but the pulse rate had stabilized at 84 bpm (Figure 2B). Repeated echocardiography demonstrated marked improvement in LV function over a short period, with an EF of 60% (Figure 3C). A small amount of pericardial effusion remained (Figure 3D, arrows). CT angiography revealed no significant stenosis in either the right or the left coronary artery (Figure 5A, 5B).

Follow-up blood tests showed an increased thiamine level of 138 ng/mL and a decreased NT-proBNP level of 673 pg/mL. During hospitalization, the patient received nutritional counseling for thiamine deficiency and was discharged on hospital day 16. He remained stable during the two-month follow-up period.

## Discussion

In this report, we described a rare but important manifestation of cardiac beriberi in a patient with a remote history of gastrectomy. The initial presentation, characterized by hypotension, rapid atrial fibrillation, and reduced LV function, closely resembled tachycardia-induced cardiomyopathy [11,12] or hyperthyroidism with significant cardiac involvement [13,14]. However, the rapid clinical improvement following thiamine administration, along with a markedly low pre-treatment thiamine level, confirmed the diagnosis of cardiac beriberi.

Although overt thiamine deficiency is uncommon in developed countries, it may occur insidiously in patients with risk factors such as chronic alcohol consumption, prolonged malnutrition, or a history of gastrointestinal surgery [8,9]. Gastrectomy can impair thiamine absorption, predisposing patients to chronic subclinical deficiency that may go unnoticed for decades. In this case, the patient had a history of gastrectomy performed 45 years earlier and reported a chronically unbalanced diet with daily alcohol consumption, indicating multiple risk factors for thiamine deficiency. Given his background, the possibility of beriberi could have been suspected at the initial presentation. This case highlights the importance for clinicians to maintain an awareness of beriberi, even in developed countries, especially when multiple risk factors are present.

The initial clinical suspicion in this case was tachycardia-induced cardiomyopathy, which shares several features with cardiac beriberi, including rapid atrial fibrillation and globally reduced LV function. Tachycardia-induced cardiomyopathy usually develops after prolonged, uncontrolled tachyarrhythmia [11,12]. With appropriate rate or rhythm control, LV function typically improves gradually over 4-12 weeks [15]. In contrast, this patient showed a rapid improvement in pulse rate, LV function, and neurological symptoms within days of thiamine administration. Given the patient’s clinical course, the globally reduced LV function at presentation was considered a premonitory or early stage of Shoshin beriberi, an acute and fulminant form of cardiac beriberi. Timely diagnosis and thiamine replacement likely averted progression to advanced hemodynamic instability.

Diagnosing cardiac beriberi is often challenging because of its rarity and non-specific symptoms. Empiric administration of thiamine serves as both a diagnostic and therapeutic approach in suspected cases. This case underscores the importance of considering cardiac beriberi in the differential diagnosis of newly diagnosed heart failure, particularly in patients with nutritional risk factors or a history of gastrointestinal surgery.

## Conclusions

In conclusion, clinicians should maintain a high index of suspicion for cardiac beriberi in patients with unexplained heart failure, particularly when neurological symptoms are present or when there is a history of malnutrition, alcohol use, or gastrointestinal surgery. Early recognition and treatment are crucial to preventing adverse outcomes. Prompt thiamine administration can lead to rapid and significant recovery, underscoring the importance of timely diagnosis.
